# Tobacco brief intervention training for chiropractic, acupuncture, and massage practitioners: protocol for the CAM reach study

**DOI:** 10.1186/1472-6882-14-510

**Published:** 2014-12-18

**Authors:** Myra L Muramoto, Amy Howerter, Eva Matthews, Lysbeth Floden, Judith Gordon, Mark Nichter, James Cunningham, Cheryl Ritenbaugh

**Affiliations:** Department of Family and Community Medicine, University of Arizona College of Medicine, 1450 N. Cherry Avenue, Tucson, AZ 85719 USA; School of Anthropology, University of Arizona, 1009 E. South Campus Drive, Tucson, AZ 85721 USA

**Keywords:** Tobacco cessation, Brief intervention, Training, Communication, Acupuncture, Chiropractic, Massage therapy, System intervention, Longitudinal study, Qualitative study

## Abstract

**Background:**

Tobacco use remains the leading cause of morbidity and mortality in the US. Effective tobacco cessation aids are widely available, yet underutilized. Tobacco cessation brief interventions (BIs) increase quit rates. However, BI training has focused on conventional medical providers, overlooking other health practitioners with regular contact with tobacco users. The 2007 National Health Interview Survey found that approximately 20% of those who use provider-based complementary and alternative medicine (CAM) are tobacco users. Thus, CAM practitioners potentially represent a large, untapped community resource for promoting tobacco cessation and use of effective cessation aids. Existing BI training is not well suited for CAM practitioners’ background and practice patterns, because it assumes a conventional biomedical foundation of knowledge and philosophical approaches to health, healing and the patient-practitioner relationship. There is a pressing need to develop and test the effectiveness of BI training that is both grounded in Public Health Service (PHS) Guidelines for tobacco dependence treatment and that is relevant and appropriate for CAM practitioners.

**Methods/Design:**

The CAM Reach (CAMR) intervention is a tobacco cessation BI training and office system intervention tailored specifically for chiropractors, acupuncturists and massage therapists. The CAMR study utilizes a single group one-way crossover design to examine the CAMR intervention’s impact on CAM practitioners’ tobacco-related practice behaviors. Primary outcomes included CAM practitioners’ self-reported conduct of tobacco use screening and BIs. Secondary outcomes include tobacco using patients’ readiness to quit, quit attempts, use of guideline-based treatments, and quit rates and also non-tobacco-using patients’ actions to help someone else quit.

**Discussion:**

CAM practitioners provide care to significant numbers of tobacco users. Their practice patterns and philosophical approaches to health and healing are well suited for providing BIs. The CAMR study is examining the impact of the CAMR intervention on practitioners’ tobacco-related practice behaviors, CAM patient behaviors, and documenting factors important to the conduct of practice-based research in real-world CAM practices.

## Background

Tobacco use accounts for nearly 443,000 deaths in the US each year, and is also responsible for $96 billion in health care expenditures [1] annually. Decades of public health tobacco control efforts have led to steady declines in tobacco use prevalence from 40.3% in 1964 to 19.3% in 2011 [2, 3]. In recent years this downward trend has been inconsistent or stagnant - prompting public health calls for new and expanded strategies for increasing tobacco cessation, including broadened insurance coverage for cessation treatment [4]. Of the 45.3 million tobacco users in the US [3], 52.4% reported in 2010 a quit attempt in the past year [4, 5]. Overall, nearly 69% of smokers report they want to quit [5]. Effective tobacco cessation treatments, recommended by the Public Health Service Guideline on Tobacco Dependence Treatment (PHS Guideline) [6], are more widely available than ever, yet are still greatly underutilized [7, 8].

Tobacco cessation brief interventions (BIs) by health care providers are clearly effective in increasing quit attempts and quit rates [6]. The PHS Guideline strongly recommends health care practitioners to provide brief behavioral interventions to encourage quitting, along with referral to professional quit coaching services such as quitlines, and cessation medications as appropriate [6].

Unfortunately, conventional practitioners have struggled to implement these PHS Guideline recommendations. In a recent Association of American Medical Colleges survey, while 86% of physicians advised patients to quit tobacco use, adherence to other PHS Guideline recommendations was far less than ideal. Only 68% of physicians assessed patient willingness to quit, 37% discussed cessation counseling options, 31% recommend nicotine replacement, 13% referred patients to others for cessation treatment, and only 7% referred patients to a quitline [9].

CAM practitioners are a significant presence in the US health care system as an increasing proportion of Americans report using CAM therapies. The 2002 National Health Interview Survey (NHIS) reported 36% of adults used some form of CAM therapy in the previous 12 months [10], a figure that had increased to nearly 40% by 2007 [11]. Another analysis of these 2007 NHIS data found that significant numbers of respondents who used CAM services in the prior year reported current smoking (17.4%) [12, 13]. Thus, CAM practitioners may see smokers regularly and have a unique opportunity to intervene. In at least one study, chiropractors were found to be more likely to engage in tobacco cessation activities than primary care physicians [14].

Despite this potential, CAM practitioners have been overlooked in the nation’s tobacco control agenda. For more than two decades, public health efforts have targeted physicians for tobacco cessation BI training [15]. Only more recently has BI training has been offered to other biomedical health care professionals (e.g. nurses, dentists, pharmacists) [15]. However, with rare exceptions tobacco cessation BI training has remained focused on conventional biomedical practitioners [16, 17]. There is a clear need to expand research on tobacco cessation training beyond conventional health practitioners to increase the potential reach and impact of brief tobacco cessation interventions.

Restricting the focus of tobacco BI training to conventional practitioners limits the potential public health impact. Unhealthy lifestyle behaviors are the root cause of the growing burden of chronic disease in the US [18–21]. Tobacco use, diet and physical activity are three lifestyle-related behaviors affecting the nation’s public health that are major modifiable health risk factors for the most prevalent chronic diseases – cardiovascular disease, cancer, cerebrovascular disease, and diabetes [22]. The Institute of Medicine report on CAM use in the US [23] found little research on the role of CAM in addressing national public health priorities requiring behavioral change. This has been recognized by the naturopathic physician and chiropractic community as well [12]. Key questions remain regarding: the role of CAM use in fostering/sustaining behavior change around tobacco use and other health behaviors; CAM practitioners’ behaviors related to promoting healthy behavior; patients’ use of CAM practitioners to support behavior change; and the potential role of CAM practitioners in preventive and promotive health if they were fully participating in a public health community of practice [24]. Tobacco cessation can serve as a model with which to examine these issues, and the CAM Reach study aims to explore these key research questions.

The purpose of the CAM Reach (CAMR) study is to develop and evaluate the effectiveness of a tobacco cessation BI training program and practice system intervention specifically adapted for chiropractors, acupuncturists, and massage therapists (CAM practitioners). The CAMR intervention is a multi-component intervention consisting of 1) a one day continuing education workshop (totaling 8 CEU credits), 2) *in situ* skills practice/skills assessment in the practitioner’s office with a practice patient, and 3) a practice system intervention to enhance practitioners’ identification and intervention with tobacco users, including ongoing practice support visits by staff members on an as needed basis. Study participants are followed for one year. The study includes both CAM practitioners and a cohort of their patients who complete surveys and interviews quarterly. We note that the CAM disciplines participating in the CAMR study customarily use different terms to refer to persons seeking their care. Chiropractors and acupuncturists usually refer to “patients”, whereas massage therapists usually say “clients”. For simplicity, we will use “patients” throughout this paper.

The specific aims of the CAM Reach study are to:

1. Evaluate the effect of the CAM Reach intervention on the primary outcomes of CAM practitioners’: conduct of tobacco use screening, brief interventions, implementation of office system changes, and referral to PHS guideline-based cessation aids. Secondary outcomes are patients’ readiness to quit, quit attempts, engagement in PHS guideline-based tobacco cessation treatments, and quit rates.
2. Explore the CAM Reach intervention’s effect on non-tobacco-using patients who want to help someone else quit, with respect to knowledge of cessation aids, and actions taken to help the tobacco user quit.
3. Conduct a qualitative study of a sub-sample of trained CAM practitioners and their patients to examine factors associated with CAM practitioners’ implementation and maintenance of cessation intervention behaviors, and changes in patients’ tobacco use.

## Methods/Design

### Main study design

The CAMR study design was a single group, one-way cross over design, with two pre-intervention and five post-intervention assessments. The CAMR intervention protocol was designed to be implemented in three waves approximately 12 months apart, (Wave 1 = chiropractors, Wave 2 = acupuncturists, and, Wave 3 = massage therapists), with a total sample of 90 practitioners, 30 of each practitioner type. The sequencing of CAM disciplines in the three waves was purposeful, in recognition that there would be differences in practice organization structure, practice patterns, and patient volume among the three CAM disciplines. This sequencing allowed for necessary adjustments in the study protocol implementation for each Wave to accommodate these differences.

Upon enrollment, practitioners completed a baseline assessment of their knowledge, attitudes, beliefs, and confidence about tobacco screening and brief interventions; and an assessment of current practice patterns related to tobacco screening. Research staff conducted a baseline assessment environmental scan of the practitioner’s practice environment and practice operations for elements related to tobacco, e.g. patient education materials, intake forms, tobacco-free environment policies, etc.). At this time research staff placed a patient cohort recruitment notice and study materials in each practice that remained in place for six months (3 months pre-training intervention and 3 months post-training). For patients who indicated interest in study participation by providing their contact information, research staff completed eligibility screening and consent over the telephone.

Three months post enrollment, practitioners participated in a one-day, in-person CAMR training workshop with a pre-test assessment immediately before the training and a post-test immediately afterwards. Approximately 1–2 weeks post training, practitioners completed a one-hour *in-situ* “practice patient” learning activity in the practitioner’s office. Practitioners completing both the workshop and the in-office practice patient learning activity were eligible for 8 Continuing Education units. All practitioners were assessed at 3, 6, 9, and 12 months after training using Computer Assisted Telephone Interviews (CATI). Patients enrolled in the longitudinal cohorts were also assessed by CATI at 3, 6, 9, and 12 months after study enrollment. The original study design was more complex, involving practice matching and cluster randomization, but early on in Wave 1, this proved to not be sustainable. Enrollment for subsequent waves began before the final data collection in the preceding wave, e.g. Wave 2 began before Wave 1 data collection was completed. All study activities took place in Tucson, Arizona and the intervention workshop was delivered at the University of Arizona campus. The University of Arizona Human Subjects Protection Program approved the study (Protocol No. 0900000349R002).

### Qualitative sub-study design

Sub-samples of practitioners and patients were invited to take part in telephone based, semi-structured, qualitative interviews. All interview guides were constructed to address domains relevant to study outcomes as well as themes that emerged during formative research with local practitioners and CAM patients.

### Study setting and participants

The CAMR study is being conducted with chiropractors, acupuncturists, and massage therapists practicing in the wider metropolitan area of Tucson, Arizona. The study also includes a cohort of patients from each practitioner.

### CAM practitioners and ancillary staff

#### Inclusion criteria

Practitioner was licensed, established in an active practice, and willing to: participate in the CAMR tobacco cessation BI training workshop and implement CAMR office system changes (e.g. tobacco user identification system, display posters, and patient handouts); adopt office procedures to administer tobacco use patient survey to facilitate patient recruitment for longitudinal patient cohort; allow ancillary staff to participate in CAMR training (staff involvement was not required), and; willing to complete study questionnaires and interviews.

#### Exclusion criteria

Practitioner was: primarily resort or spa-based and did not have an individual practice; not licensed to practice in Arizona, or; had participated in a tobacco cessation training within the preceding 2 years.

### CAM patients – tobacco users

#### Inclusion criteria

Patients were over age 18; self-identified as a tobacco user on the practitioner in-office survey, and were willing to participate in all four follow-up interviews.

#### Exclusion criteria

Patient was a seasonal resident or planned to move from the area during the study period.

### CAM patients – non tobacco users

#### Inclusion criteria

Patients were over age 18; a self-identified non-tobacco user; have a friend or family member who uses tobacco and who the patient wishes would quit, and; were willing to participate in all four interviews.

#### Exclusion criteria

Patient was a seasonal resident or planned to move from the area during the study period.

### Recruitment procedures and eligibility assessment

#### Practitioners

**Figure 1:**
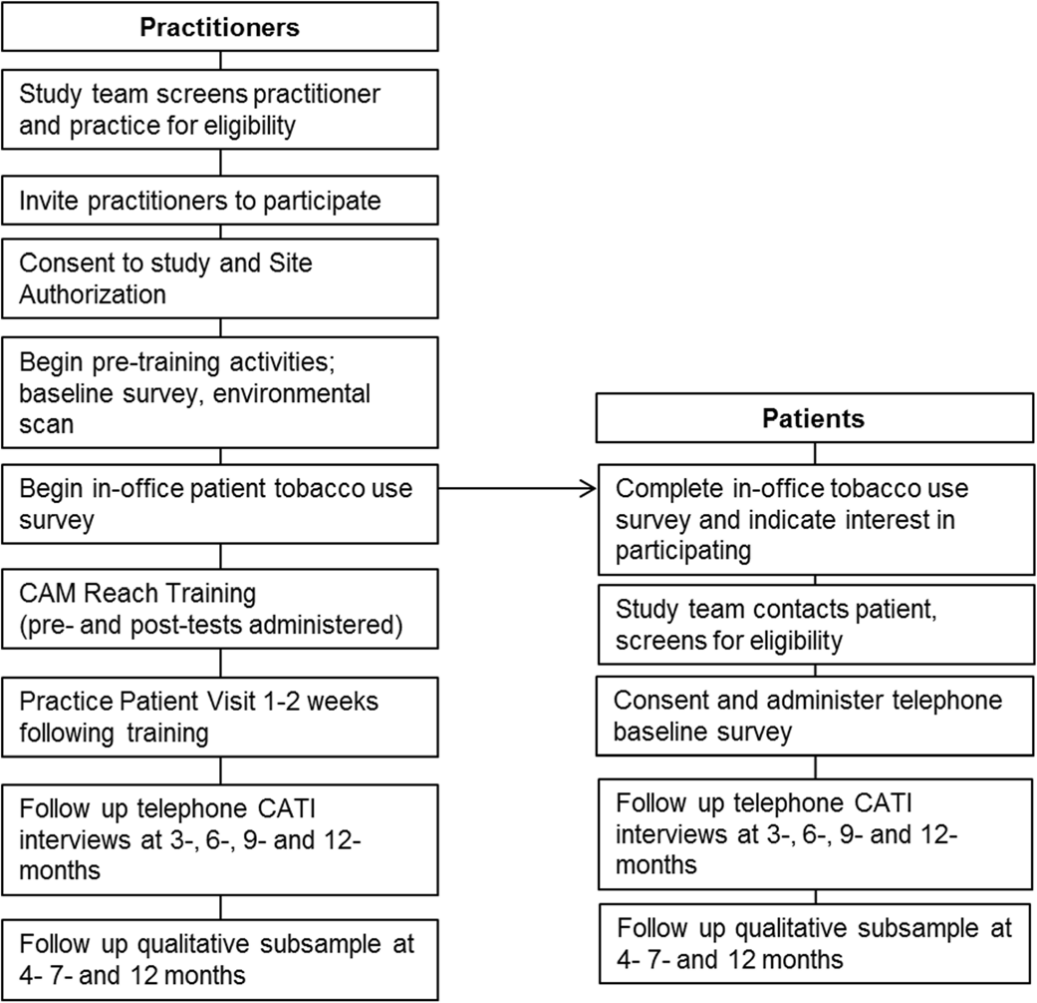
**Participant flow.**

Recruitment of practitioners began with a direct mailed letter to all currently licensed chiropractors, acupuncturists, and massage therapists in the Tucson, Arizona metro area, using address lists obtained from the Arizona state licensing boards for each discipline. The direct mailing introduced the study and informed the practitioner that they would be contacted by phone to invite their participation in the study. The follow-up phone call invited practitioners to complete a short eligibility screening survey about themselves and their practice. If practitioners were eligible and interested, an in-person visit was scheduled with study staff to review the study in detail, answer questions and obtain informed consent if the practitioner wanted to participate. Practitioners in multi-practitioner offices were asked to sign a Site Authorization Letter indicating their collective willingness for the study to take place at their site. Following written informed consent, practitioners completed baseline questionnaire assessments, and study staff performed a practice environmental scan (to document existing practice systems, policies and patient materials related to tobacco use). Distribution and collection procedures for the in-office tobacco use patient survey were also set up at this time. Figure 1 displays the study flow for participants.

### Patients

Patients were recruited for six months after practitioners enrolled in the study and completed baseline assessments. A seven item in-office survey was administered primarily as a patient identification and recruitment tool, with a single item to screen for any tobacco use in the past 7 days. The patient baseline assessment queried tobacco use history more in depth, with multiple standard items used in epidemiological surveys of tobacco use [25, 26]. Additional items regarding general health habits were included so as not to overly sensitize patients to the topic of tobacco use. A raffle prize incentive was offered for participation. Patients could fill out the survey, and provide their contact information with an indication of interest in further study participation. Study staff contacted interested patients by phone, verified their practitioner, and continued with the eligibility screening. Patients who were eligible and interested in participating were then consented over the phone and enrolled in the study. In practices where a patient saw multiple practitioners, who may or may not be participating in the study, we listed their primary practitioner as the enrolled practitioner whom they see most often. If they could not recall who their practitioner was, they were not enrolled into the study.

Client recruitment in mobile practices (Wave 3 only): Massage therapists could choose one of two methods for patient recruitment: 1) use/travel with the survey box and have project staff come empty it; or 2) have each client place completed surveys and raffle responses in individually sealed envelopes, give to practitioner to collect and mail in survey/raffle responses to research staff.

### CAMR intervention

The CAMR intervention consists of three parts: 1) an one-day training continuing education workshop, 2) *in situ* skills practice in the practitioner’s office with a practice patient, and 3) a practice system intervention to enhance practitioners’ identification and intervention with tobacco users including ongoing practice support visits by staff members on an as needed basis. It was developed after extensive formative research with the local CAM practitioner community. The CAMR intervention and the overall CAMR study itself has had ongoing input from both a local advisory panel of CAM practitioners and a national advisory panel of CAM practitioners, educators, researchers, and tobacco cessation experts.

### The CAM reach training workshop

The CAM Reach training is based on a program of research and development on tobacco cessation brief intervention training for a wide range of health professional and lay audiences [27–31], including chiropractors [16, 17]. The Helpers Program (“Helpers”) provided the foundational curriculum adapted for the CAM Reach study. Developed at the University of Arizona, Helpers is a community-based brief intervention training program that teaches lay community members, who are concerned about another’s tobacco use (i.e. “health influencers”), how to encourage quitting tobacco with a supportive “helping conversation.” Formative research revealed that CAM practitioners perceived tobacco use as a potentially sensitive topic and were concerned about potential damage to the patient-practitioner relationship if they addressed a patient’s tobacco use. Although originally intended for a lay audience, Helpers was chosen as the foundation of the training for this CAM practitioner audience because of its emphasis on encouraging quitting tobacco in ways that reduce risk of damage to social relationships. The Helpers approach differs from the traditional “5A’s” proscriptive approach recommended by the PHS Guideline and taught to conventional healthcare practitioners in that Helpers emphasizes a tobacco user-centric, motivational and non-confrontational approach to discussing tobacco use and encouraging quitting. The communication skills content in Helpers was expanded with additional content appropriate for a health professional audience that addressed motivational approaches to patient behavior change. In addition, content on health effects of tobacco use, tobacco dependence, and PHS guideline recommended cessation aids (e.g. medications, quit line referrals) were expanded to be more appropriate for a health professional audience. All training workshops were delivered at the University of Arizona.

### Practice patient role-play visit

An in-office practice patient role play assessment was scheduled 1–2 weeks following training workshop to provide practitioners the opportunity to practice and reinforce skills learned in the workshop in their own practice environment. Study staff role-play a patient scenario, provide feedback on the practitioner’s performance, and answer questions.

### CAMR practice system intervention

The CAMR system intervention combines 1) implementation of routine patient screening items on appointment documentation to identify tobacco users, 2) addition of patient education on tobacco cessation and secondhand smoke to CAM practice environment and practice building activities, and 3) receiving assistance with implementation of office system changes and periodic practice support visits throughout study engagement. Materials to support office system changes included printed intervention items: 1) a practitioner’s guide to helping patients quit tobacco; 2) patient handouts addressing topics such as: health effects of tobacco use, health effects of secondhand and third hand smoke, tobacco cessation, medications that aid in quitting, local quit-line support, referral to a training for family and friends to become support persons for tobacco users, e-cigarettes (current research evidence), and how to make a quit plan; 3) display posters to encourage patient questions regarding quitting; and 4) tobacco use documentation items including stickers with tobacco screening questions for intake forms, and chart stickers identifying tobacco using patients). Practice support/academic detailing visits were conducted as needed (every 1–2 weeks for first 3 months and then roughly every three months) and consisted of project staff visits to practitioner offices to replenish printed materials, provide newly published, relevant research articles, and to address emergent study-related questions or issues from practitioners.

### Qualitative Interviews

Interviews addressed practitioner/patient interactions, practitioner attitudes toward intervening with patients and their intervention behavior, satisfaction with training and use of training materials. The two invited sub-groups of practitioners were those who reported talking to their patients about tobacco cessation, and those who reported not talking to any patients about tobacco cessation.

Patients who reported quitting tobacco, a quit attempt, or a 50% or greater decrease in tobacco use were invited to participate in the qualitative sub-study. Interviews addressed the changes in their tobacco use and reasons for change, interactions with their CAM practitioner related to tobacco use, tobacco-related information obtained from their CAM practitioner’s office.

Patients who reported talking to their tobacco using family member or friend about quitting tobacco were also invited to participate in this part of the project. Interviews addressed the content and quality of interactions with the tobacco user about quitting, and interactions with their CAM practitioner related to tobacco, tobacco –related information obtained from their CAM practitioner’s office.

### Assessment of outcomes

Table 1 provides an overview of the schedule for assessments organized by data source. Practitioner and patient self-report questionnaires and interviews, and study staff observations were used to assess both primary and secondary outcomes of the study.

**Table 1:** **Evaluation activities, data collection time points, and domains addressed**

Assessments	Timepoint*	Domains Addressed
PRACTITIONERS		
Quantitative Questionnaire	B, 3, 6, 9, 12	Demographics (gender, race/ethnicity, age). Length of time in practice and tobacco use (baseline only); Attitudes, behavior, confidence (baseline and follow-up)
Semi-structured Qualitative Interview Guide	3, 6, 9	Patient/Practitioner interactions; Attitudes, behavior, training satisfaction, use of materials (follow-up)
CAM Reach Training Pretest	Pre	Knowledge
CAM Reach Training Post test	Post	Knowledge, confidence, training satisfaction
Practice Patient role-play	Post	Knowledge application, skills performance
PATIENTS		
Quantitative Questionnaire (Tobacco users)	B, 3, 6, 9, 12	Demographics e.g. gender, race/ethnicity, age, and tobacco use/quit history (baseline only); Attitudes and behaviors regarding tobacco cessation and CAM practitioner addressing tobacco use, patients’ tobacco use and quitting behavior (e.g. readiness to quit, quit attempts, use of PHS guideline-based cessation aids, seven-day point prevalence abstinence) (baseline and follow-up);
Quantitative Questionnaire (Non- Tobacco users)	B, 3, 6, 9, 12	All domains for tobacco users and additionally, knowledge of guideline based cessation aids, self-efficacy with helping a tobacco user quit, actions to encourage quitting (baseline and follow-up)
Semi-structured Qualitative Interview Guide	3, 6, 9	Attitudes, behavior, rationale for tobacco use status change (as applicable), use of cessation materials obtained from CAM practitioner (follow-up)
OFFICE SYSTEM		
In-office evaluation Environmental Scan and visit checklist	B, 3, 6, 9, 12	Practice environment and systems change (e.g. routine screening, use of posters, handouts); Opinions of academic detailing visits

*Baseline (B), Pre-training (Pre), Post-training (Post), 3 Months (3), 6 Months (6), 9 Months (9), 12 Months (12).

### Primary outcomes

The primary outcomes were 1) rates of screening and delivery of BIs to tobacco users by CAM practitioner, 2) CAM practitioner implementation of office system changes, and (3) CAM practitioner recommendations for PHS guideline-based tobacco cessation treatments to tobacco users.

Rates of screening and BIs by CAM practitioners in the past 90 days were measured by practitioner self-report and patient report. All CAM practitioners were asked, using a 4-point scale (“always”, “often”, “sometimes”, “never”) how often they assess tobacco use and have conversations with tobacco users about tobacco use. All tobacco-using patients who saw their practitioner since the previous assessment were asked if their practitioner talked to them about their tobacco use. Non-tobacco using patients were asked whether they had spoken to a tobacco user to encourage quitting. These assessments were performed at baseline, 3-, 6-, 9-, and 12- months. For the Wave 1 control group practitioners, the 9- and 12-month assessments occurred after the practitioners crossed over to the intervention arm and received the CAM Reach intervention. Baseline and follow-up assessments were administered by CATI to both practitioners and patients.

Implementation of office system and environmental change was measured by direct observation by the research staff during office site-visits using a pre-developed checklist. Evidence of system and environmental change were documented every three months. Indicators included the presence of tobacco patient education materials in the office environment (e.g. where and how many posters and pamphlets are being used), presence of tobacco use and second hand smoke exposure questions on standard practice intake forms or other standard screening procedures, evidence of chart flagging, and placement of CAM Reach’s patient educational materials in locations accessible to all patients. The extent to which practitioners implement office system changes was a key measure of the CAM Reach intervention’s feasibility and acceptability.

Recommendations for guideline-based tobacco cessation treatments for tobacco users were measured by practitioner self-report during the follow-up CATI’s (conducted at 3-, 6-, 9, and 12-months). Practitioners were asked what was discussed during a conversation and to which resources patients were referred. Referrals to both guideline-based (e.g. quitline, pharmacist to discuss medications) and non-guideline-based (e.g. cold laser therapy) resources were queried.

### Secondary outcomes: tobacco-using patients

Those related to tobacco users were: 1) patients’ self-reported readiness to quit, 2) quit attempts, 3) use of PHS guideline-based cessation treatments, and 4) tobacco abstinence as measured by seven-day point prevalence abstinence and prolonged abstinence. All secondary outcomes were assessed using the CATI administered patient baseline and follow up questionnaires at 3-, 6-, 9- and 12-months. Patients recruited into the study, were followed for the 12-month duration, regardless if they made a return visit to their practitioner. Questions pertinent to recent practitioner interaction were not administered if there was no practitioner visit since last assessment, but tobacco-related behavior questions were asked at each assessment.

Readiness to quit was assessed by asking current tobacco users to describe themselves as, “ thinking about quitting in the next 6 months”, “ready to quit in the next 30 days,” or “not ready to quit.” Quit attempts were assessed by asking how many times they had seriously attempted to quit smoking or using other tobacco in the last 90 days (on baseline) or since the last study interview (follow-up assessments). Quit attempts were defined as remaining abstinent for at least 24 hours with the intention of quitting. Use of PHS guideline-based cessation aids was assessed by asking the patient what methods they used when they tried to quit using tobacco. The checklist included both PHS Guideline-based recommendations (e.g. calls to quit line, use of pharmacotherapy, enrollment in internet cessation program, etc.) and non-guideline-based recommendations.

#### Tobacco abstinence measures

Nearly all tobacco users were cigarette smokers. For patients reporting abstinence, both self-reported 7- and 30-day point prevalence abstinence measures are used. Seven-day and 30-day point prevalence abstinence is defined as no tobacco use, not even a puff (or dip) for the 7 or 30 days preceding assessment. We did not include biochemical verification of tobacco abstinence, following recommendations of the SRNT Subcommittee on Biochemical Verification for studies on general populations of tobacco users, studies with low-demand characteristics, and when optimal data collection is by telephone [32].

### Secondary outcomes: non-tobacco using patients

These outcomes were self-reported actions (e.g. “helping conversations”) to encourage a tobacco user to quit. Outcomes were measured assessed at baseline and the 3-, 6-, 9-, and 12-month follow up questionnaires. Non-tobacco users were asked about the frequency of conversations with their practitioner about tobacco, the frequency of helping conversations had with tobacco users, the nature of their relationship with the tobacco user(s), and if the patient was motivated by conversations with their CAM practitioner or CAMR materials obtained from CAM practitioner’s office to talk to tobacco-using friends or family about quitting.

### Data analysis/statistical plan

#### Sample size and power

We propose to enroll 30 practitioners for each profession/wave for a total of 90 participants. The power analysis is based on the proportion of practitioners that report an outcome (e.g. performing BIs) following the intervention compared to baseline.

The primary outcome for this study is CAM practitioners’ conduct of tobacco use screening and brief interventions as measured by practitioner self-report. To our knowledge, there are no existing published studies of cessation training effects on CAM practitioner behaviors. Therefore, we are basing our effect size estimates on the published literature on cessation training for conventional biomedical practitioners.

A 2001 Cochrane review [15] examined 10 randomized controlled trials (RCTs) for effects on practitioner intervention behavior and patient cessation outcomes. Practitioners studied were: physicians, dentists, and community pharmacists. Training formats (tutorial or workshop) used the types of training method proposed for CAM Reach (lectures, videos, role plays and discussion), training contact time ranged from 1 to 8 hours. Three of the 10 trial studies included office system changes (prompts and reminders). Across all studies, trained practitioners were 1.5 to 2.5 times more likely to engage in cessation intervention behaviors. Four of the trials had training contact times/formats most similar to the CAM Reach training (2–4 hours) [33–38]. In these 4 trials, intervention practices had provider BI rates of: 54%, 64%, 66%, 31% respectively, for an average of 53.5%. Control practices had practitioner BI rates of: 31%, 44%, 27%, 10% respectively, for an average of 28%.

If we expect that 28% of practitioners will report delivering BIs at baseline and that this will increase by 25.5% so that 53.5% of practitioners report doing BIs after training, at 1-β = .80, we would need N = 26 practitioners per profession to detect a differences with α = .05. Table 2 illustrates the sample size required for a range of effect sizes and power levels.

**Table 2:** **Sample size required per profession to detect a difference in proportion at α = .05**

	Effect size		
1-β	22% (28.0% vs. 50.0%)	25.5% (28.0% vs. 53.5%)	29% (28.0% vs. 57.0%)
.75	31	23	18
.80	35	26	20
.85	41	31	24

To show the varying effect sizes we kept the pre-intervention proportion at 28% since this most closely represented the results from our pilot studies of CAM practitioners (n = 356). Twenty-nine percent (29%) of these practitioners report advising patients to quit. These data were nearly identical to the average proportion of the control group conducting BIs in the reviewed studies (29% vs. 28%). Table 2 illustrates the sample size needed to detect differences when the post-intervention proportions are both lower and higher than the reviewed studies. The target enrollment of 30 practitioners per profession will take into account potential attrition of up to 13% while still maintaining power of 80%. Additionally, this study will be well powered to detect overall differences, even of small magnitude, for the entire study population.

### Quantitative data analysis

Many of the units of analysis are nested: practitioners within CAM practitioner disciplines, time within practitioner, patients within practitioners, and time within patients, all providing the opportunity for multi-level analysis. Accordingly, hierarchical linear models will be used for most analyses, enabling assessment of change over time as well as differences between study conditions. Outcome measures (baseline and follow-up assessments nested within participants), time-invariant statistical control measures (e.g., gender, race/ethnicity), and quasi-experimental factors (pre-intervention vs. post-intervention) will be included at level 1. Higher levels will include patients (where appropriate), practitioners and disciplines. The analyses will be performed using HLM 7 Software [39]. HLM models specific to ordinal, binary and count data will be used to assess ordinal, binary and count outcome measures, respectively.

The Primary Outcome variables are practitioners’ tobacco screening behavior, BI behavior, referrals and referral to guideline-based resources. Both practitioner and patient data will be utilized. The applicable HLM model will be used, depending on whether the item was binary (yes/no) or ordinal (e.g. always, often, sometimes, never). HLM modeling will be used to assess office system changes. Tobacco cessation behavior of smoking patients will be a Secondary Outcome variable.

### Qualitative data analysis

Interviews with practitioners are semi-structured, using an interview guide developed from formative research results. Interviews focus on practitioner implementation of CAMR training/office system interventions, feedback from patients regarding provider conversations about tobacco use or tobacco-related patient materials in the office environment, and any strategies the practitioner may have developed within their practice to maintain/enhance the cessation intervention behaviors. Semi-structured interviews with patients (both tobacco users and non-tobacco users) also used an interview guide developed from formative research. These patient interviews queried about interactions with their practitioner related to tobacco (e.g. acceptability of practitioner talking to patient about tobacco use, content of the interaction) and behavioral changes promoted by the interaction(s) over time.

Interviews will be transcribed, using a verbatim transcription protocol, and analyzed using a combination of a priori (theory based) coding combined with emergent codes to produce coding-categorizing technique [40, 41]. Atlas-TI will be used for qualitative data management and coding. This strategy is a form of ethnographic content analysis that involves arranging the data into categories sorted by broader themes [42]. Codes, that can either be predetermined or emerge from the data are then attached to these categories, and are used to assign meaning to the data. Both descriptive and interpretive codes are used in this analysis with development of additional codes for emergent themes and issues [43]. We will use a standard mixed methods integrative approach to combining the qualitative, contextual data with the primary and secondary quantitative data for the final analysis.

## Discussion

The CAM Reach system intervention targets both individual CAM practitioner clinical practice behavior and the office systems that shape practice behavior. It is designed to provide chiropractor, acupuncture, and massage therapy practitioners with tobacco brief intervention skills and increase their knowledge about PHS guideline-based recommendations for tobacco cessation. Our design includes assessments from both practitioners and their patients to evaluate: 1) if and how practitioner’s self-reported behavior change and knowledge acquisition about tobacco use and cessation is perceived by their patients, and 2) if changes in practitioners’ knowledge and behavior is associated with changes in patient behavior related to tobacco.

The chosen unit for analysis is the practitioner, rather than the practice. This is an important distinction in such CAM research. For example, while the majority of the chiropractors were “solo” practitioners, seeing patients at one or more locations, and employing little or no practice support staff, a minority (6.3%) were part of multi-disciplinary practices wherein the chiropractor(s) were practicing in the same physical location with practitioners from other CAM disciplines. Other CAM practitioners at the same practice site could be employees of the lead practitioner (practice owner), or be independent practitioners subletting office space, with variable levels of patient sharing. The great heterogeneity of practice organization and business models employed by CAM practitioners poses methodological challenges for defining what constitutes a “practice” for research designs that would attempt to study whole practices. In a majority of cases, particularly with acupuncture and massage practitioners, the practitioner is the “practice”. In spite of co-location in a space, CAM practitioners tend to function independently of one another, and many practitioners operate out of one or more locations. This makes a focus on practitioners, rather than sites or practices, essential.

We implemented a single six-month recruitment period for all practitioners as described above. The 6-month patient recruitment period commenced 3 months prior to the practitioner training and continued for 3 months after practitioner training. A patient recruitment period of six months was necessary to accommodate practice patterns of the CAM practitioner sample (e.g. lower patient volume than most conventional practitioners, low ratio of new patient to return patient visits). The practitioners were then followed for 12 months based on our experience with BI training interventions [31] for non-medical health influencers(HI). Although self-efficacy tends to level off after 6 months, a significant number of HIs continued to evolve their approach to cessation interventions through accumulation of experience, seeking additional information about cessation, and interactions with other HIs. The patient cohort was also followed for 12 months to allow data collection on patterns of CAM use, and to detect delayed intervention effects with repeated point prevalence abstinence measures.

CAM practitioners are now an important presence in the US health care system, providing health and wellness care to large numbers of patients who use tobacco and/or have chronic disease(s) or major risk factors. There is a pressing need to examine the role of CAM practitioners in promoting and sustaining healthy behaviors such as tobacco cessation as members of a public health community of practice [24]. Tobacco cessation serves an ideal test case to explore this potential. Results from the CAMR study will contribute to a better understanding of the role CAM practitioners can play in public health and the possibility of forming collaborative communities of practices around health behavior change such as tobacco cessation.

## Abbreviations

BI: Brief intervention
CAM: Complementary and alternative medicine
PHS: Public health service
CAMR: CAM reach project
CATI: Computer assisted telephone interview
NHIS: National health information survey
RCT: Randomized controlled trial.
